# The prognostic value of the hypoxia markers CA IX and GLUT 1 and the cytokines VEGF and IL 6 in head and neck squamous cell carcinoma treated by radiotherapy ± chemotherapy

**DOI:** 10.1186/1471-2407-5-42

**Published:** 2005-04-25

**Authors:** Harlinde De Schutter, Willy Landuyt, Erik Verbeken, Laurence Goethals, Robert Hermans, Sandra Nuyts

**Affiliations:** 1 Department of Radiation Oncology, University Hospital Gasthuisberg, Herestraat 49, B-3000 Leuven, Belgium; 2 Department of Pathology, University Hospital Gasthuisberg, Herestraat 49, B-3000 Leuven, Belgium; 3 Department of Radiology, University Hospital Gasthuisberg, Herestraat 49, B-3000 Leuven, Belgium

## Abstract

**Background:**

Several parameters of the tumor microenvironment, such as hypoxia, inflammation and angiogenesis, play a critical role in tumor aggressiveness and treatment response. A major question remains if these markers can be used to stratify patients to certain treatment protocols.

The purpose of this study was to investigate the inter-relationship and the prognostic significance of several biological and clinicopathological parameters in patients with head and neck squamous cell carcinoma (HNSCC) treated by radiotherapy ± chemotherapy.

**Methods:**

We used two subgroups of a retrospective series for which CT-determined tumoral perfusion correlated with local control. In the first subgroup (n = 67), immunohistochemistry for carbonic anhydrase IX (CA IX) and glucose transporter-1 (GLUT-1) was performed on the pretreatment tumor biopsy. In the second subgroup (n = 34), enzyme linked immunosorbent assay (ELISA) was used to determine pretreatment levels of the cytokines vascular endothelial growth factor (VEGF) and interleukin-6 (IL-6) in serum. Correlation was investigated between tumoral perfusion and each of these biological markers, as well as between the markers mutually. The prognostic value of these microenvironmental parameters was also evaluated.

**Results:**

For CA IX and GLUT-1, the combined assessment of patients with both markers expressed above the median showed an independent correlation with local control (p = 0.02) and disease-free survival (p = 0.04) with a trend for regional control (p = 0.06).

In the second subgroup, IL-6 pretreatment serum level above the median was the only independent predictor of local control (p = 0.009), disease-free survival (p = 0.02) and overall survival (p = 0.005).

**Conclusion:**

To our knowledge, we are the first to report a link in HNSCC between IL-6 pretreatment serum levels and radioresistance *in vivo*. This link is supported by the strong prognostic association of pretreatment IL-6 with local control, known to be the most important parameter to judge radiotherapy responses.

Furthermore, the combined assessment of CA IX and GLUT-1 correlated independently with prognosis. This is a valuable indication that a combined approach is important in the investigation of prognostic markers.

## Background

Different biological and clinicopathological parameters next to the commonly used tumor and nodal stage have been proposed to help stratifying patients to certain treatment protocols. Specifically, molecular properties of the tumor microenvironment such as hypoxia, inflammation and angiogenesis are currently investigated to serve as a target for therapy[1].

The aim of the present research was to evaluate potential prognostic markers in a retrospective series of HNSCC treated by radiotherapy ± chemotherapy. A recently published report on a retrospective series of patients with a primary HNSCC treated by radiotherapy ± chemotherapy and investigated by dynamic contrast-enhanced perfusion-computer tomography (perfusion-CT), indicated that low tumoral perfusion predicted poor local control (LC), when stratified according to the median perfusion value [2]. This patient group was now used for further exploration.

Our first hypothesis was that CT-determined tumoral perfusion could be a surrogate marker for hypoxia, which is an important negative prognostic factor in radiation treatment of HNSCC [3,4].

For this purpose, we evaluated the correlation of tumoral perfusion with the expression of carbonic anhydrase IX (CA IX) and glucose transporter-1 protein (GLUT-1) using immunohistochemistry. These antigens are possible intrinsic markers of hypoxia, both mainly controlled by the hypoxia inducible factor-1 (HIF-1) pathway. Their clinical relevance to assess the expression pattern and the relation with hypoxia has been suggested by others for several tumors [5-8].

CA IX is a transmembrane carbonic anhydrase involved in the reversible hydration of carbon dioxide to carbonic acid. It is a widely investigated protein, overexpressed in many tumor types [9]. In HNSCC, its expression is associated with worse prognosis [6,10].

GLUT-1 mediates cellular glucose uptake and thus facilitates anaerobic glycolysis. This protein is largely undetectable in normal epithelium and benign tumors, but is expressed in several types of human carcinomas such as HNSCC and associated with poor prognosis [5,7,8,11].

Correlation was assessed between tumoral perfusion and each of these intrinsic markers, as well as between CA IX and GLUT-1 mutually. The prognostic value of these immunohistochemical parameters was also evaluated.

Our second hypothesis was that the CT-determined tumoral perfusion could be linked with the measurement of vascular endothelial growth factor (VEGF) and interleukin-6 (IL-6).

VEGF is a pro-angiogenic cytokine known to stimulate the proliferation and migration of endothelial cells and to promote vessel permeability. It is one of the hypoxia-responsive genes, upregulated by increased levels of HIF-1 [12,13]. VEGF serum levels are elevated in patients with HNSCC compared to healthy controls [12,14,15]. A negative prognostic role for circulating VEGF serum levels has been indicated for laryngeal carcinoma [15].

IL-6 is a proinflammatory cytokine produced by inflammatory cells as well as tumor cells [14,16]. In cancer patients, it has a multifunctional role concerning systemic alterations like cachexia as well a loco-regional impact on for example tumor cell motility and intercellular adhesion. Although mainly involved in inflammatory reactions, its expression can be induced by the transcription factor nuclear factor-kappaB (NF-κB) under hypoxic conditions [17]. Increased serum IL-6 levels have been detected in several tumor types, having a prognostic meaning for some of them [18-20]. The possible involvement of IL-6 in radioresistance was mentioned in several in vitro publications [21-23].

Available serum samples were analysed to evaluate VEGF and IL-6 at pretreatment time using an enzyme linked immunosorbent assay (ELISA).

We intended to evaluate the prognostic value of these serum cytokines, as well as the possible link between tumoral perfusion and the cytokine levels. This link could be established by the hypoxia mediated HIF-1 or NF-κB pathways.

The present work results on the possible prognostic significance of different biological and clinicopathological parameters studied in a retrospective study of patients with HNSCC. Furthermore, the relationship with CT-determined tumoral perfusion was investigated.

## Methods

### Patients and tissues

Between March 1995 and January 2000, patients with a primary HNSCC treated by radiotherapy ± chemotherapy were investigated by perfusion-CT to evaluate tumoral perfusion. The largest axial tumor diameter was selected and dynamic CT sequences were taken during injection of a contrast agent bolus. Perfusion was correlated with the carotid artery [2].

The original patient population was divided into two subgroups. For the first subgroup of 67 patients (group A), we had the disposal of formalin-fixed, paraffin-embedded biopsies, taken at the time of diagnostic endoscopy. Serial sections, 5 μm thick, were stained for hematoxylin and eosin (H&E) to allow evaluation of the representativity of the biopsy specimen, and immunohistochemically for CA IX and GLUT-1.

For the second subgroup of 34 patients (group B), we had the disposal of serum samples taken at pretreatment time and stored at -20°C until time of ELISA-analyses for VEGF and IL-6. For 24 patients of this subgroup, we had the disposal of other patient-related parameters, such as weight loss, performance status, and sedimentation rate determined at the same pretreatment time.

Histological diagnosis was made by an independent pathologist. Clinical data regarding local recurrence, regional recurrence, disease-free and overall survival were available for a minimum follow-up of four years. Patients' characteristics and treatment schedules are shown in Table 1.

### Immunohistochemistry – Group A

#### CA IX

**Figure 1:**
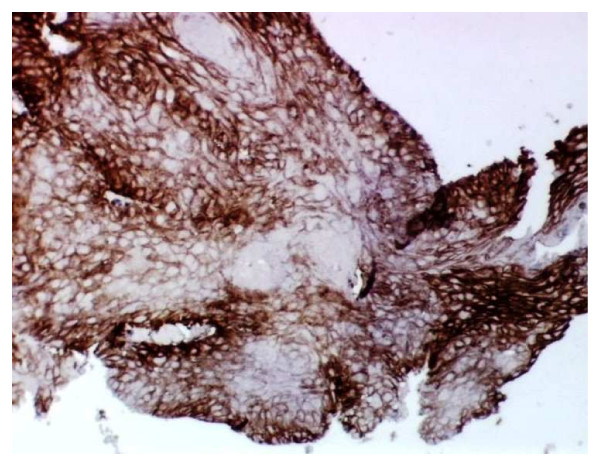
**Example of immunohistochemistry for CA IX. **Example of biopsy stained for CA IX (×100). This tumor was scored highly positive.

**Figure 2:**
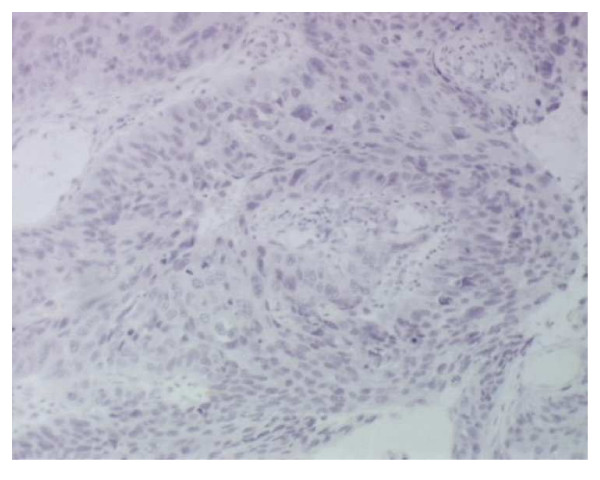
**Example of immunohistochemistry for CA IX. **Example of biopsy stained for CA IX (×100). This tumor showed a small positive percentage.

After dewaxing and rehydrating, an endogenous peroxidase block (0.3% H_2_O_2 _in methanol) was applied for 30 minutes. Slides were rinsed with Phosphate Buffered Saline (PBS) and Phosphate Buffer Saline with 0.4% Tween 20 (PBST) and then incubated with a mouse monoclonal anti-human CA IX antibody (M75, 1/50 dilution in PBS; Bayer, USA) [24] for 30 minutes at room temperature. After 3 × 5 min rinses in PBST, incubation with a secondary anti-mouse labeled polymer/horse radish peroxidase conjugate solution (HRP, 30 minutes at room temperature) was carried out before rinsing 3 more times. Visualization was performed by diaminobenzidine (DAB) substrate (DAKO, Substrate Chromogen System). After rinsing in PBS, sections were counter-stained with Harris hematoxylin (Merck). Slides were then dehydrated and mounted with "micromount mounting medium" (Surgipath 03731). Substitution of the primary antibody with PBS was used as a negative control. To exclude nonspecific binding of the primary antibody, we performed the same protocol using an irrelevant antibody of the same species (monoclonal mouse anti-human Smooth Muscle Actin, DAKO, 1/100 dilution). This showed no binding (data not shown). As a positive control, we used normal gastric mucosa. Representative examples of positive and negative staining results are shown in Figure 1 and Figure 2, respectively.

#### GLUT-1

**Figure 3:**
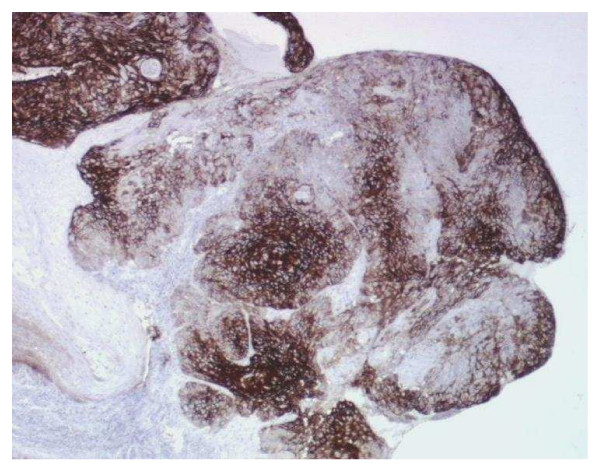
**Example of immunohistochemistry for GLUT-1. **Example of biopsy stained for GLUT-1 (×40). This tumor was scored highly positive.

**Figure 4:**
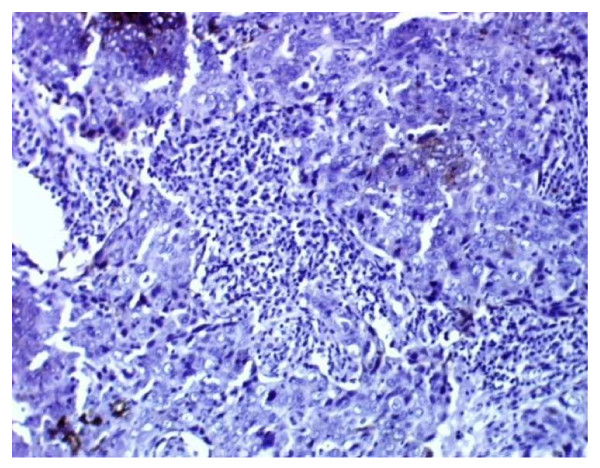
**Example of immunohistochemistry for GLUT-1. **Example of biopsy stained for GLUT-1 (×40). This tumor showed a small positive percentage.

After dewaxing and rehydrating, antigen retrieval was performed by microwaving sections immerged in 10 mM citrate buffer (pH 6). After cooling down, sections were rinsed with Tris Buffered Saline, pH 7,5 with 0.1% Tween 20 (TBST) and an endogenous peroxidase block (3% H_2_O_2 _in destilled water) was applied for 15 minutes. Slides were rinsed with TBST and incubated with "Protein Block Serum Free" (DAKO) for 20 minutes to block nonspecific binding of immunoglobulins to the tissue. In a next step, sections were incubated with a polyclonal rabbit anti-human GLUT-1 antibody (DAKO, 1/200 dilution in TBS) at 4°C overnight. After 3 × 5 minutes rinses with TBST, incubation with a secondary anti-rabbit labeled polymer/HRP conjugate solution (30 minutes at room temperature) was carried out before rinsing 3 more times. Visualization, counter-staining, dehydrating and mounting was done as for the CA IX staining. Omitting of the primary antibody served as a negative control. To exclude nonspecific binding of the primary antibody, we performed the same protocol using an irrelevant antibody of the same species (polyclonal rabbit anti-human S-100, DAKO, 1/300 dilution). This showed no binding (data not shown). Erythrocytes, present in every section, served as internal positive control for GLUT-1. Representative examples of positive and negative staining results are shown in Figure 3 and Figure 4, respectively.

#### Quantification technique

All morphologic investigations were performed by the same investigator, who was blinded to the clinical and follow-up data.

The representativity of the biopsies was evaluated on the H&E slices. All the tissue figuring in the biopsy was classified into one of the following compartments: viable invasive tumor cells, carcinoma in situ, normal epithelium, necrotic tissue, blood vessels, collagenous connective tissue, muscle tissue, inflammatory connective tissue, and a "residu". The proportion of each of these compartments was measured by means of a point counting system. At an optical magnification of 100 × and using a grid with 10 matched points, at least 100 points per tissue section were counted as hitting one or another compartment. The proportion of points fitting each compartment yielded directly the volume proportions of the corresponding compartments.

CA IX and GLUT-1 immunostained sections were semiquantitatively scored. The fraction of cells stained was expressed as a percentage of the total tumor area, both fractions being estimated by light microscopic inspection of between 1 and 25 low power fields (×40) per patient.

### Serum VEGF and IL-6 immunoassay – Group B

ELISA kits for specific cytokines (VEGF and IL-6) were used according to the manufacturer's protocol (Quantikine Human VEGF/IL-6 Immunoassay, R&D Systems, Minneapolis, MN). Serum samples were incubated in duplicate on microtiter plates coated with a specific monoclonal antibody for 2 hours at room temperature. The plates were washed to remove unbound antibody. After incubation of a conjugate solution, a substrate solution was added. Color development was stopped after 20–25 minutes, depending on the assay. A microplate reader was used to determine colorimetric densities at 570 nm and 450 nm for each sample. The readings at 570 nm were subtracted from those at 450 nm to get the optical density for each sample. Final results were calculated from a standard curve generated by a form parametric logistic curve fit. Results were expressed in pg/ml.

### Statistics

The association between discrete and continuous variables was tested by the Mann Whitney's U-test or Kruskall Wallis test as appropriate. Correlations between continuous variables were obtained using Spearman's rank correlation. Survival curves were constructed using the Kaplan-Meier method, all time intervals being calculated from the date of the first radiotherapy session. Individual factors were evaluated for their predictive value by the log rank test. The Cox proportional hazard model was used for multiple regression analysis. All tests were two-sided, using a significance level of 0.05. All statistics were done using Statistica software 6.

## Results

### Group A: immunohistochemistry

#### Representativity of the biopsies

On average, the biopsies were composed of viable tumor cells (52.4%, range 9.76%–92.13%), carcinoma in situ (1.3%), normal epithelium (2.0%), necrotic tissue (2.8%), blood vessels (6.0%), collagenous connective tissue (17.8%), muscle tissue (1.4%), inflammatory connective tissue (14.0%), and a "residu" (mucous glands i.e.; 2.8%). Because of their high tumor content the biopsies were considered as representative to score tumoral expression of CA IX and GLUT-1.

#### Biological and clinicopathological parameters

The CT-determined perfusion rate ranged from 12.4 ml/min/100 g to 274.4 ml/min/100 g with a median value of 87.9 ml/min/100 g (n = 67). The percentage of CA IX positivity ranged from 0 to 87.8% with a median of 17.14%, and the GLUT-1 from 0 to 100% with a median of 67.5%. Both stainings were almost exclusively membranous, except for some aspecific cytoplasmatic CA IX staining.

There was no significant correlation between the CT-determined tumoral perfusion and percentage positivity for CA IX or GLUT-1.

We also found no significant correlation between tumor characteristics such as tumor stage, nodal stage and differentiation on one hand and perfusion-CT, CA IX and GLUT-1 percentages on the other hand.

#### Correlation with outcome

In univariate analysis, the prognostic value of different clinicopathological and biological parameters towards local control (LC), regional control (RC), disease-free survival (DFS) and overall survival (OS) was evaluated.

The tumor stage (T3-4 vs. T1-2) predicted a poorer RC (48.7 vs. 73.3% at 2 years; p = 0.03) and OS (43.7 vs. 64.6% at 5 years; p = 0.002). N3 stage also seemed to predict poorer outcome (LC: 9.8 vs. 44.8% at 2 years, p = 0.04; RC: 17.6 vs. 62.7% at 2 years, p = 0.0009; DFS: 0 vs. 37.9% at 5 years, p = 0.03; OS: 0 and 56.7% at 5 years, p = 0.002). No significant correlation was found between tumor differentiation and outcome.

Poorer tumor perfusion stratified according to the median value was linked with poorer LC (33.2 vs. 48.2 % at 2 years, p = 0.03) and DFS (26.4 vs. 38.6% at 5 years, p = 0.03).

For the CA IX and GLUT-1 percentages we could not find a significant correlation with outcome. Nevertheless, a trend towards poorer LC (31.4 vs. 44.6% at 2 years p = 0.08) and DFS (27.8 vs. 34.7% at 5 years, p = 0.08) was seen in patients with CA IX as well as GLUT-1 values above the median.

The multivariate analysis included tumor stage (T3-4 vs. T1-2), nodal stage (N0-1-2 vs. N3), differentiation grade, perfusion (≥ median) and combined CA IX and GLUT-1 percentages (CA IX and GLUT-1 ≥ median).

As shown in Table 2, the combined CA IX and GLUT-1 percentage was an independent prognostic factor for LC (p = 0.02) and DFS (p = 0.04) with a trend for RC (p = 0.06). N3 stage was a strong independent predictor of LC (p = 0.006), RC (p = 0.0001), DFS (p = 0.0001) and OS (p = 0.0002). Higher tumor stage predicted poorer overall survival (p = 0.006). The perfusion showed no clear correlation with LC (p = 0.09).

### Group B: serum immunoassay

#### Biological and clinicopathological parameters

The CT-determined tumoral perfusion rate ranged from 29.6 ml/min/100 g to 263.6 ml/min/100 g with a median value of 88.7 ml/min/100 g (n = 34). IL-6 values ranged from 0.4 pg/ml to 57.3 pg/ml with a median of 5.4 pg/ml. VEGF values ranged from 73.5 pg/ml to 1613.1 pg/ml with a median of 326.9 pg/ml.

**Figure 5:**
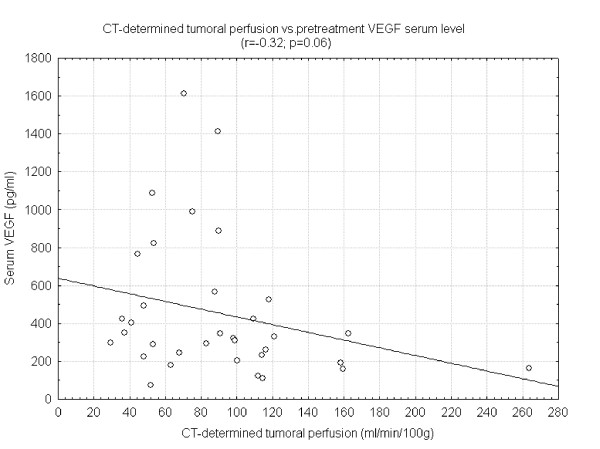
**Correlation between CT-determined tumoral perfusion and pretreatment VEGF serum levels. **There was a trend for poorer CT-determined tumoral perfusion to correlate with higher pretreatment VEGF serum levels (r_S _= -0.32, p = 0.06; n = 34).

There was no significant correlation between the perfusion-CT measurements, IL-6 and VEGF values. However, Figure 5 shows that the perfusion was borderline inversely related to VEGF (p = 0.06) suggesting lower perfusion being associated with higher serum VEGF levels.

Patients with higher nodal stage showed significantly more elevated IL-6 serum levels (p = 0.04). Other tumor characteristics such as tumor stage and differentiation grade were not correlated with either perfusion-CT, serum VEGF or serum IL-6 levels.

#### Correlation with outcome

In univariate analysis, the prognostic value of IL-6, VEGF and clinicopathological parameters towards LC, RC, DFS and OS was evaluated.

**Figure 6:**
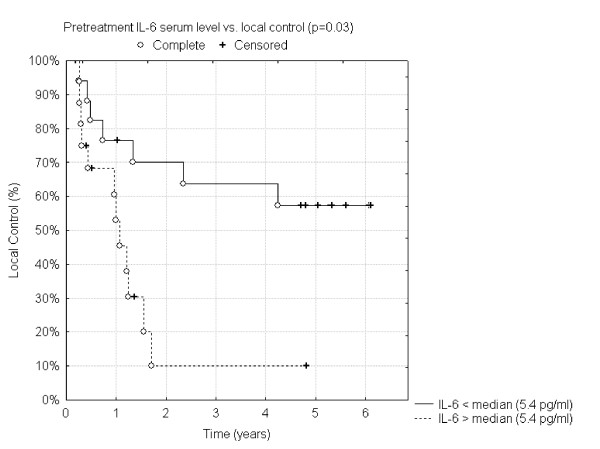
**Kaplan-Meier curve local control analysis. **There was a significant difference in local control between patients with pretreatment IL-6 serum levels above and below the median (9.3 vs. 70.4% at 2 years, p = 0.03).

**Figure 7:**
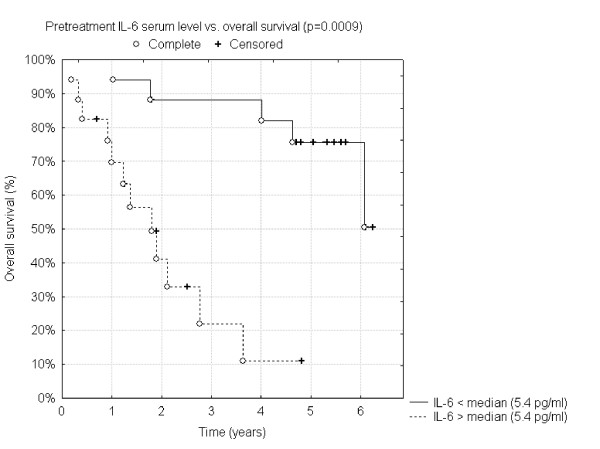
**Kaplan-Meier curve overall survival analysis. **There was a highly significant difference in overall survival between patients with pretreatment IL-6 serum levels above and below the median (11.9 vs. 74.9% at 5 years, p = 0.0009)

As clear from Figures 6 and 7, the IL-6 serum level, stratified according to the median, was found to have a predictive value towards LC (9.3 vs. 70.4% at 2 years, p = 0.03), DFS (8.6 vs. 57.7% at 5 years, p = 0.05) and OS (11.9 vs. 74.9% at 5 years, p = 0.0009). Tumor stage, nodal stage and tumoral perfusion were found to have only a borderline prognostic value, probably due to the small sample size in this group (data not shown). We found no correlation between pretreatment cytokine serum levels and other patient-related parameters such as weight loss, performance status and sedimentation rate determined at the same pretreatment time.

The multivariate analysis included tumor stage (T3-4 vs. T1-2), nodal stage, perfusion (≥ median), VEGF serum level (≥ median) and IL-6 serum level (≥ median). Table 3 shows that the IL-6 serum level was the only independent predictor of LC (p = 0.009), DFS (p = 0.02) and OS (p = 0.005).

## Discussion

The tumor microenvironment plays a critical role in tumor aggressiveness and treatment response. Several microregional features have been shown to be relevant for treatment outcome, such as hypoxia, inflammation and angiogenesis. The major question stays whether these markers can be used in an integrated way to stratify patients to certain treatment options [1].

Starting from a retrospective series of patients with HNSCC treated by radiotherapy ± chemotherapy, we investigated the prognostic significance of several biological and clinicopathological parameters. Earlier established results showed that for this whole patient group, CT-determined tumoral perfusion was correlated with poorer LC [2].

Two subgroups were now used for further analysis. In the first subgroup of 67 patients, immunohistochemistry for CA IX and GLUT-1 was performed on a representative paraffin-embedded pretreatment biopsy. In the second subgroup of 34 patients, ELISA was used to determine pretreatment VEGF and IL-6 serum levels. The correlation between these parameters mutually, as well as their prognostic value, was investigated.

Hypothesizing that CT-determined tumoral perfusion could be an inverse marker for hypoxia (less perfusion relates to more hypoxia), we assessed the correlation between tumoral perfusion, the intrinsic hypoxia markers CA IX and GLUT-1, and the serum levels of VEGF and IL-6, known to be upregulated under certain microenvironmental conditions like hypoxia.

Besides a borderline inverse association between perfusion and VEGF, no significant correlation was found in our study. There are three possible explanations in support of this finding.

First, it is likely that the dynamic CT measures rather acute hypoxia, while CA IX and GLUT-1, as well as VEGF and IL-6, are more indicative for chronic hypoxia.

Second, the CT-determined tumoral perfusion in this patient group was a measurement that took place through one region of interest, i.e. the maximum diameter of the tumoral region. On the other hand, CA IX and GLUT-1 immunohistochemistry was performed on one tumor biopsy taken at the periphery of the tumor field, and VEGF and IL-6 values were measured as systemic serum levels.

Third, the relationship between perfusion and oxygenation has shown to be in a precious balance [1,25].

The borderline association found between perfusion and VEGF can be explained through the hypoxia-hypothesis. Nevertheless, an opposite finding would also have been possible, associating the angiogenetic property directly with perfusion.

The further analysis of the biological and clinicopathological parameters showed that patients with higher nodal stage expressed significantly higher IL-6 serum levels. This experience is in accordance with previous reports linking elevated IL-6 levels to extensive disease [20], and more specifically, to lymph node involvement [26]. It is not surprising since this multifunctional cytokine plays an important role in promoting tumorigenesis.

Concerning the prognostic value of the clinicopathological parameters, we found a correlation with outcome for known factors like tumor stage (RC and OS) and N3 stage (LC, RC, DFS, OS). Of these, only the nodal stage appeared to behave as an independent prognostic factor for RC and DFS.

Concerning the prognostic value of the biological parameters, poorer tumor perfusion predicted poorer LC and DFS, as expected [2]. This correlation was lost in the smallest subgroup, probably due to a too small patient number in this group. Furthermore, in multivariate analysis, the perfusion only kept a borderline prognostic value for outcome.

For CA IX and GLUT-1, the combined assessment of patients with both markers expressed above the median did indicate an independent correlation with worse LC, RC and DFS. This result demonstrates that a combined assessment gives additive information, though we have to note that the prognostic value of each of both markers is not clearly established, given prognostic [5-8,27] as well as non-prognostic [28] publications.

In the second subgroup, IL-6 pretreatment serum level stratified according to the median seemed to be the only independent predictor of LC, DFS and OS. VEGF pretreatment levels were not correlated with prognosis.

The prognostic value of IL-6 has been described in many tumor types [18], but not yet in HNSCC treated by radiotherapy ± chemotherapy. In our multivariate analysis, we found a strong prognostic impact of the IL-6 pretreatment serum level on local control. This might indicate that there is indeed a link between IL-6 and radioresistance, as already shown *in vitro *by others [21-23].

VEGF- reports in literature are a bit conflicting, with papers demonstrating [15] and denying [29] a prognostic role for VEGF serum levels at pretreatment time.

In this study, due to methodological reasons, we performed serum and immunohistochemical analyses for two subgroups instead of the whole patient group.

The advantage of using serum samples over immunohistochemistry on biopsies is that the ELISA test is quickly and simple to perform. Furthermore, the technique is not too invasive and quite established.

On the other hand we must be aware that HNSCC cells may not be the only source of the elevated serum cytokine levels in patients. The serum levels may also depend in part upon individual host inflammatory responses and conditions like cachexia, and certainly not upon hypoxia only. In our patient group however, we did not see a correlation between VEGF or IL-6 serum levels and sedimentation rates, determined at the same pretreatment time. Furthermore, there are some pitfalls in the measurement of these cytokines [30]. Concerning VEGF, the isoform specificity and platelet interaction may be of influence. The use of banked frozen serum for analysis may also be perceived as a shortcoming. Because of the retrospective character of this study, we could not control the methods used for taking and storing the serum samples.

## Conclusion

In conclusion, we found a prognostic value in this series of patients with HNSCC treated by radiotherapy ± chemotherapy for some of the investigated parameters, with a major role for IL-6. To our knowledge, we are the first to report a link in HNSCC between IL-6 pretreatment serum levels and radioresistance *in vivo*. This link is supported by the strong prognostic association of pretreatment IL-6 with local control, known to be the most important parameter to judge radiotherapy responses. This finding has to be validated in other, preferably prospective, studies.

Furthermore, the combined assessment of CA IX and GLUT-1 correlated independently with prognosis. This is a valuable indication that a combined approach is important in the investigation of prognostic markers.

We believe that additional to the standard TNM classification this combined assessment of several biological and clinicopathological parameters will show most appropriate to stratify patients to certain treatment protocols. The efforts made to search for prognostic factors in radiotherapy settings will stretch towards a more efficient combination of radiotherapy with other treatment strategies like chemotherapy, radiosensitizers, bioreductive drugs and biological targeting. Therefore, further research will be necessary.

## Competing interests

The author(s) declare that they have no competing interests.

## Authors' contributions

HDS carried out the immunohistochemistry, ELISA's and the acquisition and analysis of data. She also drafted the manuscript. WL participated in the conception of the study and helped to draft the manuscript. EV assisted in the immunohistochemistry performance and scoring technique. LG participated in the scoring process, and the analysis of data. RH performed perfusion-CT and collected data. SN conceived of the study, participated in its design and coordination and helped to draft the manuscript. All authors read and approved the final manuscript.

## Pre-publication history

The pre-publication history for this paper can be accessed here:



## Figures and Tables

**Table 1:** Patient characteristics, tumor characteristics and treatment modalities

		**Group A (n = 67)**	**Group B (n = 34)**
**Age (years)**	mean (min-max)	56 (36–82)	58 (42–79)

**Sex**	M	60	31
	F	7	3

**cT stage**	T1	2	1
	T2	17	13
	T3	27	7
	T4	21	13

**cN stage**	N0	15	11
	N1	12	3
	N2	31	17
	N3	9	3

**Tumor site**	glottic	3	2
	supraglottic	16	10
	hypopharynx	20	7
	oropharynx	24	14
	oral cavity	2	0
	nasopharynx	2	1

**Radiotherapy**	66 Gy/ 2 Gy	20	4
	70 Gy/1.4 Gy	4	1
	70 Gy/2 Gy	41 (2+ cisplatinum)	28 (1+cisplatinum)
	80.5 Gy/1.15 Gy	2 (1+cisplatinum)	1 (+ cisplatinum)

Patient characteristics, tumor characteristics and treatment modalities are shown for Group A and Group B, both subgroups of the original study of CT-determined tumoral perfusion in patients with HNSCC treated by radiotherapy ± chemotherapy. Cisplatinum was given at a dose of 100 mg/m^2 ^on days 1, 22 and 43.

cT: clinical tumor stage. cN: clinical nodal stage. M: male. F: female. Gy: Gray.

**Table 2:** Multivariate analysis for Group A

	**Local Control**			**Regional Control**			**Disease-free Survival**			**Overall Survival**		
**Parameter**	** RR **	**95% CI**	**P value**	** RR **	**95% CI**	**P value**	** RR **	**95% CI**	**P value**	** RR **	**95% CI**	**P value**

T1-2 vs. T3-4	1.21	0.58–2.53	0.62	2.74	0.99–7.62	**0.05**	1.40	0.70–2.83	0.34	3.85	1.46–10.18	**0.006**
N 0-1-2 vs. N3	3.75	1.47–9.60	**0.006**	7.68	2.72–21.70	**0.0001**	5.70	2.34–13.85	**0.0001**	6.05	2.32–15.77	**0.0002**
Differentiation	1.50	0.90–2.49	0.12	1.64	0.89–3.02	0.11	1.49	0.93–2.40	0.12	0.81	0.45–1.47	0.49
Perfusion > median	0.55	0.28–1.09	0.09	1.07	0.46–2.51	0.87	0.61	0.32–1.15	0.12	0.65	0.48–0.88	0.28
GLUT-1 & CA IX > median	2.33	1.12–4.82	**0.02**	2.24	0.97–11.58	0.06	2.06	1.05–4.07	**0.04**	1.53	0.65–3.60	0.33

Multivariate analysis (Cox proportional hazard model) for Group A towards local control, regional control, disease-free survival and overall survival. Significant P values are marked in bold. RR: relative risk. CI: confidence interval.

**Table 3:** Univariate and multivariate analysis for pretreatment IL-6 serum levels (Group B)

	**Local control**			**Regional control**			**Disease-free survival**			**Overall survival**		
	
	**RR**	**95% CI**	**P value**	**RR**	**95% CI**	**P value**	**RR**	**95% CI**	**P value**	**RR**	**95% CI**	**P value**
Univariate	0.98	1.39–9.78	**0.03**	1.29	0.37–4.53	0.68	3.24	1.25–8.50	**0.05**	8.30	2.44–28.50	**0.0009**
Multivariate	4.06	1.40–11.70	**0.009**	1.80	0.40–8.17	0.45	3.39	1.22–9.39	**0.02**	7.61	1.82–31.80	**0.005**

Univariate and multivariate analysis (Cox proportional hazard model) for IL-6 (> or < median) towards local control, regional control, disease-free survival and overall survival. Significant P values are marked in bold. RR: relative risk. CI: confidence interval.
